# Optimization of β-Carotene Extraction from Tucumã Fruit (*Astrocaryum aculeatum*) Using Ionic Liquids: Evaluation of Efficiency, Thermal and Light Stability

**DOI:** 10.1021/acsomega.5c11477

**Published:** 2025-12-18

**Authors:** Anne Caroline Gouvêa Ferreira, Bruna Ribeiro de Lima, Wallice Luiz Paxiúba Duncan, Leandro Pereira França, Jaime Paiva Lopes Aguiar, Francisca das Chagas do Amaral Souza

**Affiliations:** † Laboratory of Functional Analysis and Food Chemistry, 191073 National Institute for Amazonian Research (INPA), Manaus, Amazonas 69067-375, Brazil; ‡ Postgraduate Program in Agriculture in the Humid Tropics, National Institute for Amazonian Research (INPA), Manaus, Amazonas 69067-375, Brazil; § Laboratory of Functional Morphology, Federal University of Amazonas (UFAM), Manaus, Amazonas 69077-000, Brazil

## Abstract

This study aimed to optimize the extraction of β-carotene from the pulp of tucumã (*Astrocaryum aculeatum*), an Amazonian fruit rich in bioactive compounds, using the ionic liquid [C_4_mim]­[BF_4_] as a sustainable alternative to conventional acetone extraction. Using a central composite design, the optimal extraction conditions were determined: a solid-to-liquid ratio of 1:5, 10 min of extraction time, and 65.5 W of power. The [C_4_mim]­[BF_4_] method achieved a higher yield (12.10 mg/100 g) than acetone (8.75 mg/100 g), with greater thermal and photolytic stability. In an oily medium at 90 °C, the extract exhibited a half-life of 3466 min, and colorimetric analysis indicated reduced color degradation, confirming its potential as a stable natural colorant. Toxicity tests revealed low toxicity in all samples against *Artemia salina* larvae, with [C_4_mim]­[BF_4_] showing no significant lethal effects, whereas the acetone extract exhibited higher activity (LC_50_ = 682.32 μg/mL) and the positive control, lapachol, displayed high toxicity (LC_50_ = 33.51 μg/mL). Predictive PASS analysis indicated that β-carotene and α-carotene have high probabilities of antioxidant, anti-inflammatory, and free radical scavenging activities, while lutein demonstrated anti-inflammatory, antihepatotoxic, and cardioprotective potential. In all cases, Pa values exceeded Pi values, confirming the pharmacological feasibility of the compounds. Thus, [C_4_mim]­[BF_4_] is a green, efficient, and safe alternative for the extraction and stabilization of carotenoids from tucumã, with potential applications in the food, cosmetic, and pharmaceutical industries.

## Graphical abstract

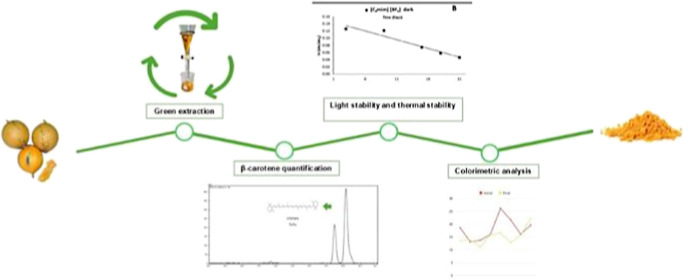

## 1 Introduction

The Amazon region harbors one of the greatest diversities of organisms and ecosystems on the planet. It is estimated that approximately 10% of the world’s vertebrate and plant species are concentrated in an area that accounts for about 0.5% of the Earth’s total surface. The Amazon ecosystem contains around 30% of all vascular plant species.^1^ Due to their wide distribution and diverse uses, palms are considered one of the most important botanical families. However, in order for them to be better utilized, further studies are needed to demonstrate yet unknown benefits, new uses and, subsequently, their incorporation into agriculture.^2^

Despite their natural wealth, many of these fruits remain underexplored.^3^ Among these species, the tucumã (*Astrocaryum aculeatum*) stands out, a palm tree from the Arecaceae family that, although it has great potential, is still underutilized. Endemic to Brazil, it is predominant in the northern region, in the states of Acre, Amazonas, Rondônia and Roraima, and it also occurs in the central-western region, in the state of Mato Grosso. *Astrocaryum vulgare* is not endemic to Brazil. It occurs in the northern region, in the states of Amapá, Pará and Tocantins; in the northeast, in the state of Maranhão, and in the central-west, in Goiás, with a predominance in the eastern portion of the Amazon region, especially in the state of Pará, which is the possible center of origin and diversity of the species.^4^ Its palms can reach heights of 10 to 25 m, have spiny trunks, and show a great capacity for adaptation to poor and degraded soils.^5^ The fruits of the tucumã are a rich source of essential nutrients and bioactive compounds, which are particularly known for their high content of natural pigments, especially carotenoids, with an emphasis on β-carotene as the main component.^6,7^

β-Carotene is an essential precursor of vitamin A, crucial for eye, immune and cellular health.^8^ In addition to its role as a provitamin, it is known for its antioxidant properties, which help strengthen the body’s defenses and prevent cellular aging.^9,10^ Its use has sparked growing interest in the food industry as a viable alternative to synthetic dyes, which are often associated with potential risks and environmental impacts.^11^ Natural colorants, such as β-carotene, have been gaining popularity due to their lower toxicity and the positive effects associated with their consumption.^12^

However, the application of natural pigments in the food industry still faces challenges, such as the use of traditional extraction methods with organic solvents, which have significant limitations due to volatility, toxicity and their environmental impact.^13^ In response, “green” extraction techniques have been gaining prominence, with improvements and expanded usage.^14^ Among these techniques, ionic liquids have proven to be an efficient and sustainable approach for extracting bioactive compounds.^15^ Defined as salts with relatively low melting points (usually below 100 °C), ionic liquids are composed of ionic species that include bulky and asymmetric organic cations, as well as inorganic or organic anions with delocalized charge.^16^ The interest in its use has grown due to its versatility, distinct physicochemical properties, and ease of processing, which has led to a focus on multidisciplinary research.^17^ This class of compounds is considered an alternative to volatile solvents due to its high capacity to dissolve a wide variety of substances, including organic, inorganic, and organometallic compounds, as well as biological molecules and metal ions.^18^

In comparison with other emerging green solvents, such as deep eutectic solvents (DES) and natural deep eutectic solvents (NADES), ionic liquids are relatively better understood and more extensively studied.^19^ Although DES/NADES are recognized for their low toxicity and biodegradability, their high viscosity can limit mass transfer, reducing the efficiency of extracting highly hydrophobic compounds such as carotenoids. In contrast, ionic liquids offer tunable polarity, generally lower viscosity in many formulations, and a greater solvating ability for nonpolar molecules. These characteristics allow for more effective cell disruption, in addition to enabling their recovery and reuse.^20,21^

When compared with conventional extraction methods, ionic liquids also present relevant advantages, such as higher extraction capacity, thermal stability, and resistance to degradation. Additionally, being less polluting, nonvolatile and environmentally safe, they reduce the risk of contamination both for food and in the environment.^22^ In addition to these characteristics, the extraction process using ionic liquids contributes to CO_2_ capture, biomass fractionation, and metal removal, offering environmentally friendly alternatives to conventional solvents.^23^

The use of ionic liquids in the extraction of bioactive compounds from Amazonian fruits has shown efficiency for extracting carotenoids, with the application of ultrasound techniques leading to an increased total carotenoid extraction yield from the fruits when compared to conventional extraction with acetone.^24^ Ionic liquids are efficient solvents, widely used in the pretreatment and fractionation of lignocellulosic materials, in cellulose dissolution, in the conversion into chemicals, and in the extraction of bioactive compounds. In the case of extractions using ionic liquids combined with probe sonication, a favorable performance is observed, accelerating material dissolution without the need for external heating and reducing processing time from 12 h to just 40 min. This extraction process, based on cavitation, breaks down cell walls, facilitating the penetration of the ionic liquid, allowing complete dissolution, and promoting changes in the physicochemical characteristics of the materials. This technique represents an advancement over conventional methods, making the process faster and more economically viable.^25^

Although the number of available cationic and anionic species is limited, the ionic liquids most commonly used in extraction processes feature large asymmetric cations, based on imidazolium and pyridinium, combined with small and diffuse inorganic anions such as tetrafluoroborate (BF_4_^−^) and hexafluorophosphate (PF_6_^−^). The ionic liquid [C_4_mim]­[BF_4_] shows good extraction yields and high efficiency in obtaining β-carotene from fruits. In addition to proven extraction efficiency, ionic liquids exhibit thermal and light stability, indicating their potential for use in the dye industry under various environmental conditions, thus reinforcing their role as green solvents for the food industry with low environmental impact.^26–28^

Aligned with the choice of solvent in the bioactive compound extraction process, the optimization of experimental conditions plays a crucial role in maximizing yield and extract quality. In this regard, design of experiments (DOE) has been widely used as a robust statistical tool for developing efficient and reproducible processes. Through this approach, it is possible to systematically analyze the variables involved and identify the optimal conditions, ensuring a more sustainable and effective process in preserving the properties of bioactive compounds.^29–31^

Therefore, this study aimed to optimize the method of β-carotene extraction from tucumã (*A. aculeatum*) pulp, using ionic liquid as an alternative to traditional organic solvents. The most efficient solvent was selected based not only on extraction yield but also on its compatibility with carotenoids, considering factors such as thermal stability, light resistance and color intensity.

## 2 Materials and Methods

### 2.1 Raw Materials and Chemicals

The fruits of *A. aculeatum* were acquired in the municipality of Autazes, state of Amazonas, Brazil. The fruits of *A. aculeatum* were acquired in the municipality of Autazes, state of Amazonas, Brazil, in August 2024. The selected materials were sanitized with sodium hypochlorite and water. After this process, they were washed with running water to remove any potential contaminants. The pulp was then removed, frozen at −80 °C, freeze-dried and stored at −40 °C to preserve the material for future analyses.

The ionic liquid 1-butyl-3-methylimidazolium tetrafluoroborate ([C_4_mim]­[BF_4_]) with a purity of >99% and the β-carotene standard (purity >99%) were purchased from Sigma-Aldrich. Acetonitrile, ethyl acetate, HPLC-UV-grade ethanol, petroleum ether (purity 100%) and diethyl ether (purity 100%) were purchased from Merck.

### 2.2 β-Carotene Extraction from Tucumã Pulp (*A. aculeatum*)

#### 2.2.1 Conventional Extraction with Acetone

The β-carotene extraction with acetone.^32^ To 1.0 g of freeze-dried fruit pulp, 70 mL of acetone (100%) was added. The obtained β-carotene was transferred to a solution of petroleum ether: ethyl ether (2:1, w/w). The upper phase, consisting of ether and the carotenoid, was dried using a rotary evaporator at <37 °C. The dry material was then stored at −80 °C until further analysis.

#### 2.2.2 Alternative Extraction with Ionic Liquid

The extraction using the ionic liquid was adapted from the methodology.^27^ The ionic liquid used was 1-butyl-3-methylimidazolium tetrafluoroborate ([C_4_mim]­[BF_4_]). The ionic liquid solution was initially prepared with ethanol at a 1:1 (w/w) ratio. The solid−liquid ratio (S/L) of fruit/solvent and the extraction time (min) in the ultrasonic bath (Eco-Sonics, Ultronique) were optimized using a design of experiments (DoE) approach. After extraction, the samples were filtered and the β-carotene was purified using thermal precipitation at −80 °C. To recover the remaining carotenoid in the ionic liquid, 10 mL of ethanol was added to the solution. After clarifying the ionic liquid solution, it was stored at −80 °C until further analyses.

### 2.3 Experimental Design to Maximize β-Carotene Extraction

The optimization of the extraction process was conducted using design of experiments (DoE). For this, a central composite rotational design (CCRD) was employed, aiming to determine the ideal extraction conditions for β-carotene from *A. aculeatum* using [C_4_mim]­[BF_4_]. The applied CCRD (2^3^) evaluated the solid−liquid ratio (S/L) of fruit/solvent (*X*_1_), extraction time (*X*_2_) and power (*X*_3_), with three central points, resulting in 17 treatments (Table 1). The statistical calculations during the optimization phase, including model fitting, coefficient significance (*p* < 0.05) and analysis of variance (ANOVA), were performed using the Protimiza Experimental Design software (Protimiza Experimental Design, Brazil).

**Table 1:** CCRD (2^3^) Applied for Optimizing the Extraction of β-Carotene Present in the Pulp of *Astrocaryum aculeatum*

run	*R* (S/L) (*X*_1_)	time (*X*_2_, min)	power (*X*_3_, W)	β-carotene^a^ (*Y*, area)
1	−1 (1:2)	−1 (5)	−1 (45)	40,348
2	1 (1:4)	−1 (5)	−1 (45)	212,646
3	−1 (1:2)	1 (15)	−1 (45)	62,377
4	1 (1:4)	1 (15)	−1 (45)	184,554
5	−1 (1:2)	−1 (5)	1 (86)	10,874
6	1 (1:4)	−1 (5)	1 (86)	194,736
7	−1 (1:2)	1 (15)	1 (86)	18,941
8	1 (1:4)	1 (15)	1 (86)	124,613
9	−1.68 (1:1.32)	0 (10)	0 (65.5)	52,976
10	1.68 (1:4.68)	0 (10)	0 (65.5)	215,353
11	0 (1:3)	−1.68 (1.59)	0 (65.5)	9696
12	0 (1:3)	1.68 (18.41)	0 (65.5)	43,633
13	0 (1:3)	0 (10)	−1.68 (31.02)	51,151
14	0 (1:3)	0 (10)	1.68 (99.98)	125,021
15	0 (1:3)	0 (10)	0 (65.5)	125,842
16	0 (1:3)	0 (10)	0 (65.5)	140,008
17	0 (1:3)	0 (10)	0 (65.5)	145,000

a Intensity measured using HPLC-PDA at λ_max_ of 450 nm.

### 2.4 Identification and Quantification of β-Carotene

The obtained extract was identified and quantified for β-carotene content in triplicate, using a high-performance liquid chromatograph (Shimadzu, LC20AT) with the following specifications: 10 μL automatic injector loop; VP-ODS column with spacer (150 × 4.6 mm, 5 μm); and UV–visible diode array detector (Shimadzu, SPD-M20A). The mobile phase consisted of an elution gradient of acetonitrile: ethyl acetate: water (88:2:10) to (85:15:0) for 15 min, maintaining this ratio for an additional 30 min, with a flow rate of 1.0 mL per minute at 29 °C.^31^ The chromatograms were processed at 450 nm and the spectra were obtained between 200 and 600 nm.

The presence of β-carotene was confirmed by comparing the retention time and UV–visible profile with the standard. Quantification was performed using the standard calibration curve (0.04 to 0.5 mg/g, *y* = 3,000,000*x* − 110,346; *R* ^2^ = 0.9937). The curve was composed of six points (in duplicate) of concentrations versus the area of the respective chromatographic peak, using Microsoft Office Excel 2016 (Microsoft Corp.) and the statistical data were verified using Minitab18 software.

The concentrations of the substance extracted with [C_4_mim]­[BF_4_] were compared to those extracted with acetone through a series of statistical tests. Initially, the Shapiro–Wilk test was applied to verify the normality of the model residuals. Subsequently, the homogeneity of the variances between the groups was assessed using Levene’s test. Next, the mean concentrations of the extracts with [C_4_mim]­[BF_4_] and acetone were compared using the *t*-test for independent samples. In the light stability and thermal stability assays, the statistical analyses also followed student’s *t*-test, adopting a 95% confidence level. The comparison between the means and their respective standard deviations was conducted considering the different media (aqueous and oily) as well as the solvents used (acetone and [C_4_mim]­[BF_4_]).

### 2.5 Evaluation of β-Carotene Stability

The β-carotene-rich extracts obtained by extraction with acetone and [C_4_mim]­[BF_4_] were subjected to stability analysis, following the methodology.^33^ The degradation of β-carotene was assessed using a spectrophotometer (Shimadzu, UV mini 1240), with the extracts subjected to two conditions: oily and aqueous. In the oily medium, the solution was prepared using 60 mL of sunflower oil and the extract until an absorbance condition of 1.0 at 450 nm was achieved. For the aqueous medium, a solution of 10 μg/mL of extract was prepared with a final volume of 60 mL of ethanol/water solution (20:80). The assays were performed in triplicate. The evaluation was carried out in terms of degradation constant (*K*_d_) values. *K*_d_ values are determined by the slope of the line when plotted as the ln of the final absorbance (abs) divided by the initial absorbance (abs_0_) as a function of time. The half-life (*t*_1/2_) (eq 1) was also evaluated; this parameter is measured by the time it takes for the initial compound to reduce to 50% of its absorbance.

1 $$t_{1/2}=\frac{-\ln0.5}{K_{d}}$$

#### 2.5.1 Light Stability

The materials were subjected to two different conditions: light exposure and absence of light. In the light-exposed medium, two 40 W LED lamps were installed, positioned 9 cm from the samples, with direct incidence on the materials. In the dark medium, the samples were placed in glass containers and stored in a light-protected area to avoid any external interference. The evaluations were carried out twice a week, with spectrophotometer readings taken over a thirty-day period.

#### 2.5.2 Thermal Stability

The samples were subjected to two treatments at temperatures of 60 and 90 °C in a water bath for a period of 8 h, with aliquots of the material taken every 20 min for analysis in the spectrophotometer.

### 2.6 Color

The color of the samples was measured using a colorimeter (HunterLab). The color parameters were lightness (L*), red-green chromaticity (a*) and yellow-blue chromaticity (b*). L* represents brightness (luminosity, ranging from −100 to +100), a* represents redness to greenness (−60 to +60 chroma), b* represents yellowness to blueness (−60 to +60 chroma) and Δ*E* represents the distance between two colors in the L*, a*, b* color space. The fruit extracts were evaluated in both aqueous and oily media using a device calibrated at 25 °C. From the obtained values, the hue angle was calculated and expressed in degrees (°hue). The solid color angles start at 0° for red (+a*), 90° for yellow (+b*), 180° for green (−a*) and 270° for blue (−b*). The calculations were performed using Excel software following the methodology.^34^

### 2.7 Test Toxicity

To evaluate the toxicity of the samples, a lethality bioassay using *Artemia salina* was employed. This assay is widely recognized as a reliable tool for assessing the toxicity of various substances due to its simplicity, rapid response, and low cost, which support its extensive application in toxicological studies.^35^ The toxicity of the crude extracts was assessed following the methodology previously described,^36^ with slight modifications.

For larval hatching, 100 mg of *A. salina* eggs were added to a glass aquarium containing a 35% saline solution (35 g of synthetic sea salt dissolved in 1 L of distilled water) and maintained under artificial illumination (incandescent lamp) at 28 °C for 48 h. After hatching, 10 nauplii were transferred into test tubes containing the extracts at concentrations of 1000, 500, 250, 100, 50, and 25 mg/mL, and incubated under the same temperature and lighting conditions for 24 h.

All assays were performed in triplicate for each concentration. Lapachol was used as the positive control, while 1% DMSO served as the negative control. Toxicity was determined by counting the number of dead larvae (immobile individuals) after 24 h of exposure.^37^ LC_50_ and LC_90_ values were calculated by probit analysis using appropriate statistical software.

### 2.8 Pass Prediction

Research focused on evaluating the biological activities of compounds is often discontinued before reaching the final stages of development due to side effects, severe adverse reactions, or unknown toxicity. In this context, PASS (*Prediction of Activity Spectra for Substances*) emerges as a promising tool based on structure−activity relationships, developed to predict the biological activity spectrum of a substance. With an average accuracy above 90%, PASS can simultaneously predict hundreds of pharmacological effects and biochemical mechanisms, making it extremely useful in the early stages of drug discovery. Its application significantly contributes to the preliminary screening of compounds with therapeutic potential and guides subsequent experimental studies.^38,39^

In accordance with the literature, a compound is considered to possess potential biological activity when its Pa (probability of activity) value exceeds 0.70.^40^ In the present study, three carotenoids were selected as targets due to their reported biological relevance. However, PASS analysis was performed only for β-carotene, the predominant compound in the sample, while α-carotene and lutein were included as references based on literature data to support comparative interpretation.^41^

## 3 Results and Discussion

### 3.1 Optimization of β-Carotene Extraction from *A. aculeatum* with [C_4_mim]­[BF_4_]

The CCRD results for the three variables studied in the optimization of the [C_4_mim]­[BF_4_] extraction are presented in Table 1. At a 95% confidence level, factors *X*_1_ and *X*_2_ showed a significant influence on the β-carotene area (*p* < 0.05). The mathematical model obtained to describe the relationship between the variables and the response is represented by eq 2

2 $$Y_{1}=128,811.99+62,759.22x_{1}-31,635.01x_{2}^{2}$$

The statistical significance of the model was verified through analysis of variance (ANOVA). The regression was highly significant (*p* = 0.00001), indicating that the fitted equation statistically and robustly explains the variability of the experimental data. The coefficient of determination (*R* ^2^ = 0.8065) shows that the model is capable of explaining approximately 80.65% of the observed variability in the response. Furthermore, the lack-of-fit analysis resulted in a *p*-value of 0.0717, suggesting that the fitted equation is suitable for describing the response within the experimental range studied.

In Figure 1, the surface and contour response graphs generated from eq 2 are shown. It demonstrates that a high concentration of β-carotene occurs when the *R* (S/L) ratio is at its maximum level and the treatment time is at the central point. Therefore, the ideal conditions to maximize the β-carotene content from *A. aculeatum* are obtained with an *R* (S/L) ratio of 1:5, a treatment time of 10 min and a power of 65.5 W. Thus, the predicted optimal condition was tested in triplicate and this condition was used in the quantification step to compare with the acetone extraction presented in the next section.

**Figure 1:**
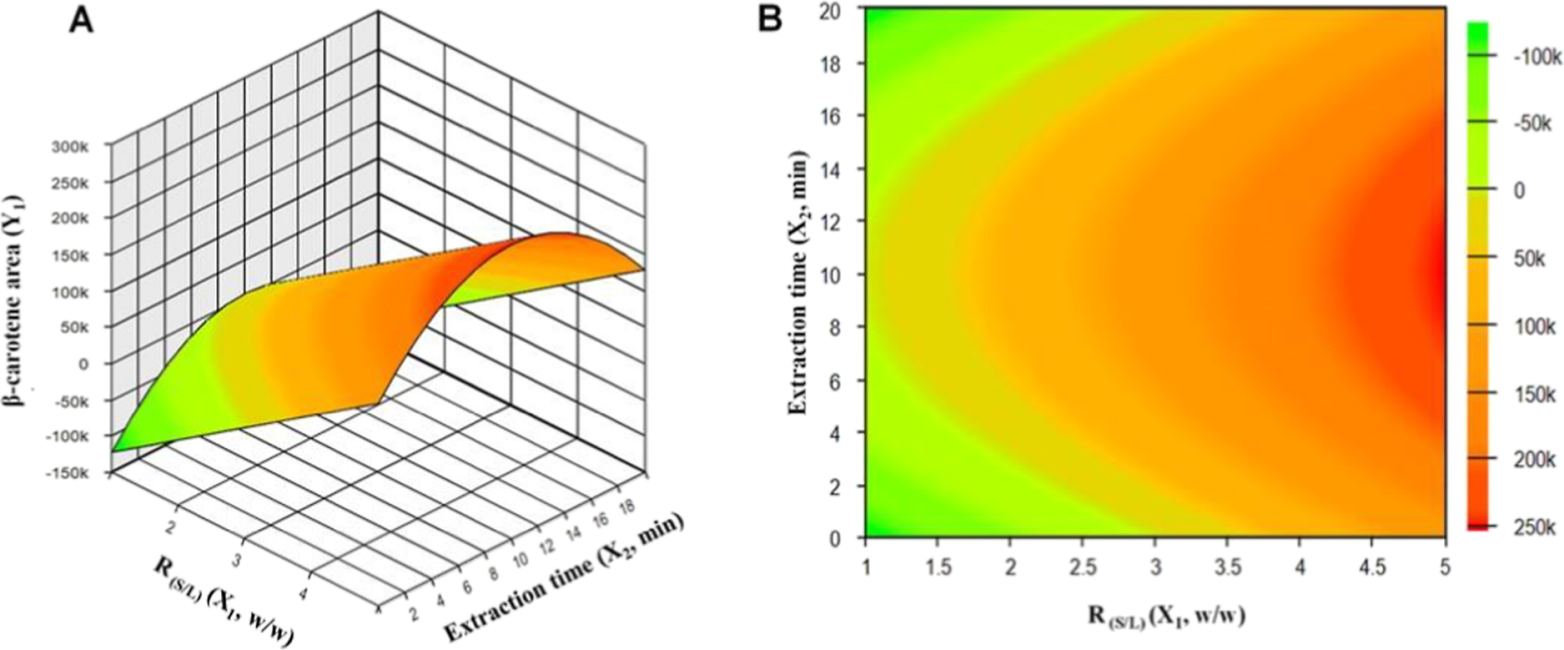
Response surface (A) and contour plot (B) for the optimization of β-carotene content in tucumã (*Astrocaryum aculeatum*).

### 3.2 Determination and Quantification of β-Carotene Obtained from Tucumã (*A. aculeatum*) Pulp Using HPLC-DAD

The analyzed extracts demonstrated the presence of β-carotene, as shown in Figure 2, when compared with the standard, which confirms the results reported in the literature. β-Carotene is widely recognized as the main carotenoid present in the pulp of *A. aculeatum* as highlighted.^32,42^ Fruits from palms, especially those with yellow-orange pulp, are abundant sources of carotenoids, with β-carotene being the most prominent, positioning Amazonian fruits among the best plant sources of provitamin A.^43^

**Figure 2:**
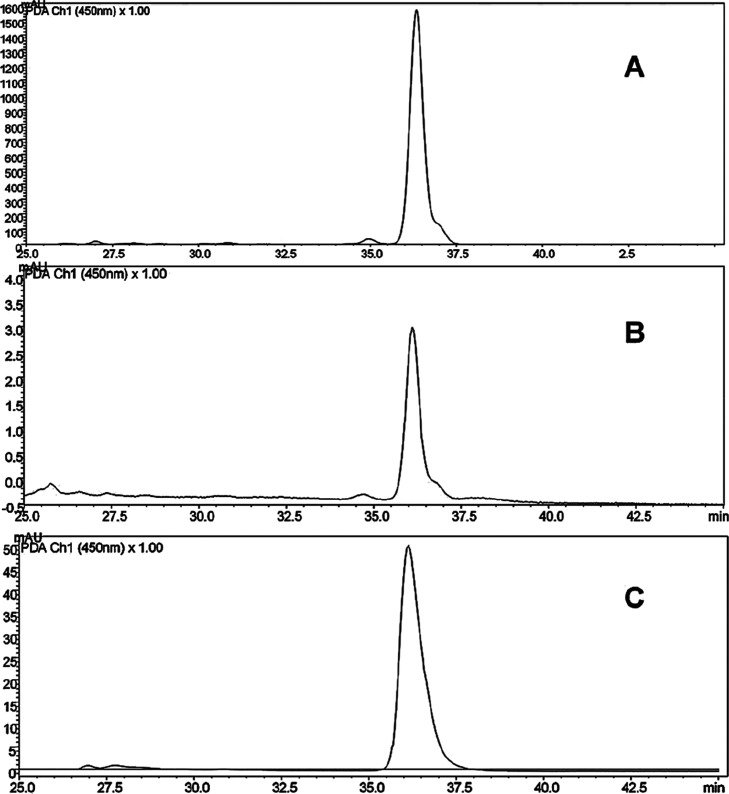
Representative HPLC-DAD chromatograms at 450 nm from tucumã (*Astrocaryum aculeatum*) pulp extraction (A) extraction with [C_4_mim]­[BF_4_]; (B) extraction with acetone; (C) β-carotene standard.

Figure 3 shows that the extraction of β-carotene with [C_4_mim]­[BF_4_] was significantly more effective than with acetone. The efficiency of extraction with ionic liquids can be explained by their ability to break down cell walls and facilitate the solvation of the target compound. Their structural interactions, such as hydrogen bonding, π–π interactions and van der Waals forces, play a crucial role in the extraction of bioactive compounds, with the possibility of adjusting their properties by modifying the cations and anions.^44,45^ These characteristics, combined with their high chemical and thermal stability, allow ionic liquids to maintain the integrity of bioactive compounds during extraction, minimizing their degradation.^46^

**Figure 3:**
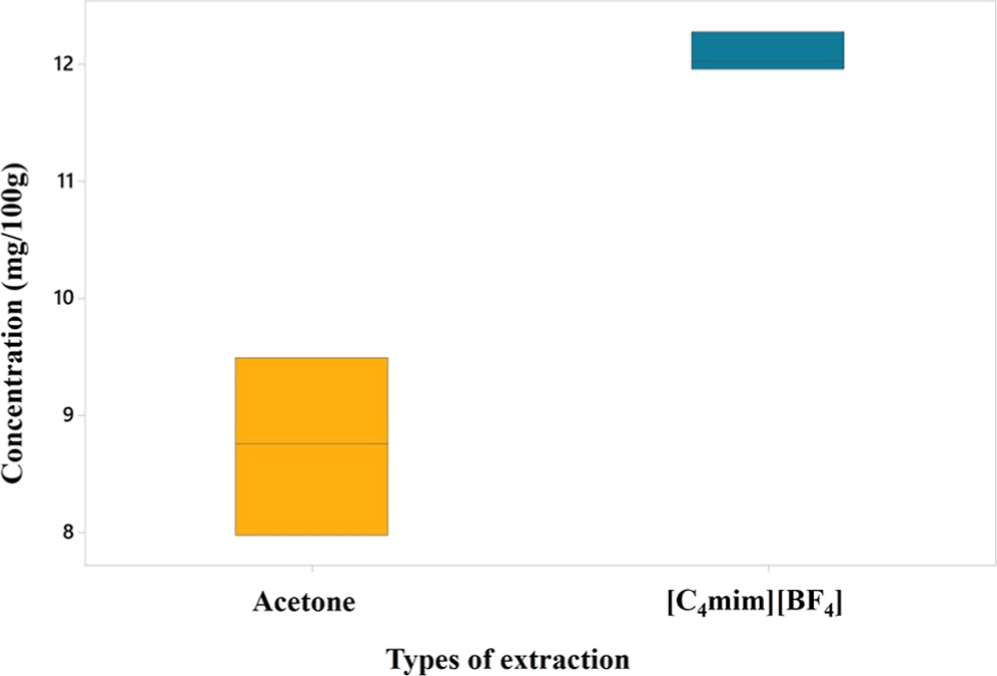
β-Carotene content in tucumã (*Astrocaryum aculeatum*) pulp.

In contrast, traditional β-carotene extraction methods that use petroleum-derived solvents face challenges such as compound degradation due to oxidation by heat and light, as well as causing environmental impacts and toxic waste risks.^45–48^ In this context, ionic liquids emerge as promising alternatives, allowing solvent reuse and making the process more efficient and sustainable.^24,27,28,33^

In the study conducted, the extraction of tucumã with [C_4_mim]­[BF_4_] yielded a value of 12.10 mg/100 g of β-carotene, while the extraction with acetone resulted in 8.75 mg/100 g. In comparison, the study reported a value of 4.7 mg/100 g of β-carotene.^49^ While found 56.7 mg/100 g for the same compound.^42^ These values can be attributed to several variables that affect extraction, such as pre- and postharvest conditions, plant genotype, ripening stage, cultivation type, climatic conditions and the processing method used.^50^

### 3.3 Light and Thermal Stability of the β-Carotene-Rich Extract Obtained from Tucumã (*A. aculeatum*) Pulp

The stability of the extracts was evaluated in two commonly used media in the food industry: aqueous and oily media, as they are frequently present in food formulations.^33^ The choice of these systems aims to simulate different matrices and understand the behavior of carotenoids under conditions that mimic real applications. Table 2 presents the degradation constant (*K*_d_) and half-life (*t*_1/2_) values of the β-carotene-rich extracts subjected to different environmental stress conditions.

**Table 2:** Degradation Constant (*K*_d_) and Half-Life (*t*_1/2_) the β-Carotene-Rich Extract Obtained from Tucumã (*Astrocaryum aculeatum*) Pulp^a^

light stability		light		dark	
medium	solvent	*K*_d_ (days)	*t*_1/2_ (days)	*K*_d_ (days)	*t*_1/2_ (days)
aqueous	acetone	0.0442 ± 0.01^a^	16 ± 2.44^a^	0.0677 ± 0.00^a^	10 ± 0.39^a^
	[C_4_mim][BF_4_]	0.0177 ± 0.00^b^	39 ± 10.16^b^	0.0110 ± 0.00^b^	63 ± 8.75^b^
oily	acetone	0.0519 ± 0.00^a^	13 ± 0.10^a^	0.0119 ± 0.00^a^	58 ± 0.69^a^
	[C_4_mim][BF_4_]	0.0330 ± 0.00^b^	21 ± 2.00^b^	0.0074 ± 0.00^b^	94 ± 5.91^b^

thermal stability		60 °C		90 °C	
medium	solvent	*K*_d_ (min)	*t*_1/2_ (min)	*K*_d_ (min)	*t*_1/2_ (min)
aqueous	acetone	0.0012 ± 0.00^a^	578 ± 26.39^a^	0.0019 ± 0.00^a^	372 ± 11.70^a^
	[C_4_mim][BF_4_]	0.0008 ± 0.00^a^	866 ± 15.58^a^	0.0015 ± 0.00^a^	454 ± 15.73^a^
oily	acetone	0.0018 ± 0.00^a^	397 ± 0.00^a^	0.0018 ± 0.00^a^	393 ± 13.08^a^
	[C_4_mim][BF_4_]	0.0015 ± 0.00^a^	462 ± 17.98^a^	0.0002 ± 0.00^b^	3466 ± 0.00^b^

a Values with different letters (a–b) in the same evaluated medium (oil and aqueous) are significantly different (*p* < 0.05).

Regarding light stability, the extracts obtained with [C_4_mim]­[BF_4_], both in aqueous and oily media, showed lower degradation constant (*K*_d_) values and longer half-life (*t*_1/2_) times, indicating greater resistance of the carotenoids to photodegradation (Figures S1 and S2). Furthermore, the extracts kept in the dark exhibited higher half-life (*t*_1/2_) times, supporting studies that indicate exposure to light accelerates carotenoid degradation.^51^

The thermal evaluation also showed that [C_4_mim]­[BF_4_] provided greater stability to the carotenoids in both media and at the temperatures tested (Figures S3 and S4). Notably, the oily medium at 90 °C, for which the extract exhibited a significantly longer half-life (3466 min), indicating high thermal protection. These findings attribute the ability to stabilize bioactive compounds under extreme conditions to ionic liquids, reinforcing their potential in the extraction and preservation of natural pigments.^52^ These findings attribute the ability to stabilize bioactive compounds under extreme conditions to ionic liquids, reinforcing their potential in the extraction and preservation of natural pigments.

In general, the data indicate that [C_4_mim]­[BF_4_] was more efficient in preserving carotenoids, both under light exposure and at elevated temperatures, particularly in the oily medium at 90 °C. This behavior reinforces the potential of this ionic liquid as a green alternative for the extraction and stabilization of natural pigments. The stabilizing profile observed aligns with the literature, which has already shown that ionic liquids can confer greater stability to carotenoids even under adverse conditions, such as light exposure and high temperatures.^11,27,31^ Due to their favorable properties, ionic liquids have proven to be attractive in various relevant chemical processes, including catalysis, biocatalysis, synthetic chemistry and electrochemistry. Among their main advantages are low viscosity, negligible or zero vapor pressure under environmental conditions, adjustable solubility, high thermal stability and low corrosivity. Furthermore, these solvents do not pose the risks associated with conventional organic solvents, representing a lower environmental impact.^53,54^

### 3.4 Colorimetry of Light and Heat Stability of β-Carotene-Rich Extracts Obtained from Tucumã (*A. aculeatum*) Pulp

The results obtained through colorimetric analysis are shown in Table 3. These results highlight the changes in color characteristics (L*, a*, b* and ΔE*) in different systems (aqueous and oily media), solvents (acetone and [C_4_mim]­[BF_4_]) and environmental conditions (exposure to light and temperature) during the thermal and light stability tests. Statistical analysis was specifically applied to the Δ*E* parameter, which represents the total color difference and therefore encompasses the other chromatic parameters. The statistical comparison considered the different media used and the solvents applied in the experiments.

**Table 3:** Colorimetry of the β-Carotene-Rich Extract Obtained from Tucumã (*Astrocaryum aculeatum*) Pulp^a^

light stability			initial				final				
medium	environment	solvent	L*	a*	b*	ΔE*	L*	a*	b*	ΔE*	Δ*E* differential
aqueous	light	acetone	73.50 ± 0.7	−0.53 ± 0.0	3.56 ± 0.2	18.6 ± 0.5	68.15 ± 1.6	−0.75 ± 0.0	2.72 ± 0.4	13.49 ± 1.5	−5.13 ± 2.11^a^
		[C_4_mim][BF_4_]	67.80 ± 0.2	−0.52 ± 0.0	4.91 ± 0.1	13.1 ± 0.1	66.51 ± 0.0	−0.78 ± 0.1	9.17 ± 0.6	13.86 ± 1.7	0.72 ± 1.71^b^
	dark	acetone	68.42 ± 0.2	−0.67 ± 0.0	3.68 ± 0.1	13.7 ± 0.1	65.01 ± 0.2	−0.53 ± 0.0	7.42 ± 0.0	11.07 ± 0.2	−2.65 ± 0.33^a^
		[C_4_mim][BF_4_]	70.72 ± 0.1	−0.68 ± 0.0	5.23 ± 0.1	16.0 ± 0.1	70.48 ± 0.0	−0.63 ± 0.0	5.70 ± 0.0	15.81 ± 0.1	−0.21 ± 0.10^b^
oily	light	acetone	59.24 ± 0.3	−0.10 ± 0.9	2.64 ± 0.8	26.1 ± 0.7	69.36 ± 2.9	−0.26 ± 0.3	10.8 ± 1.1	16.56 ± 1.9	−9.58 ± 0.01^a^
		[C_4_mim][BF_4_]	66.88 ± 3.2	3.31 ± 1.5	21.96 ± 0.3	21.7 ± 0.2	66.57 ± 3.2	−0.86 ± 0.5	8.20 ± 1.3	12.83 ± 1.4	−8.95 ± 0.32^a^
	dark	acetone	65.01 ± 0.4	3.07 ± 0.1	16.40 ± 0.5	16.1 ± 0.6	63.51 ± 0.1	2.96 ± 0.1	16.9 ± 0.2	15.71 ± 0.2	−0.40 ± 0.89^a^
		[C_4_mim][BF_4_]	65.55 ± 1.0	2.58 ± 0.1	20.25 ± 0.8	19.6 ± 1.2	66.29 ± 0.6	2.91 ± 0.2	23.1 ± 1.5	22.39 ± 1.6	2.79 ± 0.43^b^

thermal stability			initial				final				
medium	temperature	solvent	L*	a*	b*	ΔE*	L*	a*	b*	ΔE*	Δ*E* differential
aqueous	60 °C	acetone	66.98 ± 0.1	0.28 ± 0.0	11.74 ± 0.1	14.5 ± 0.1	69.06 ± 0.2	−0.41 ± 0.0	4.71 ± 0.0	14.31 ± 0.1	−0.24 ± 0.18^a^
		[C_4_mim][BF_4_]	64.90 ± 0.4	0.68 ± 0.0	9.76 ± 0.2	11.7 ± 0.2	66.23 ± 0.1	0.05 ± 0.0	7.20 ± 0.0	11.96 ± 0.1	0.24 ± 0.27^a^
	90 °C	acetone	69.76 ± 0.7	−0.80 ± 0.0	3.86 ± 0.1	15.0 ± 0.7	71.56 ± 0.1	−0.97 ± 0.4	4.07 ± 0.2	16.83 ± 0.1	1.79 ± 0.74^a^
		[C_4_mim][BF_4_]	68.73 ± 0.1	−0.78 ± 0.0	3.34 ± 0.0	14.0 ± 0.0	67.39 ± 0.3	−0.73 ± 0.0	3.71 ± 0.0	12.73 ± 0.4	−1.31 ± 0.44^b^
oily	60 °C	acetone	64.54 ± 1.0	2.48 ± 0.1	11.50 ± 0.1	12.2 ± 0.9	62.44 ± 0.5	2.69 ± 0.1	16.9 ± 0.5	15.16 ± 0.7	2.93 ± 0.24^a^
		[C_4_mim][BF_4_]	64.94 ± 0.7	1.74 ± 0.2	15.39 ± 1.9	15.3 ± 1.9	63.03 ± 1.7	1.91 ± 0.7	16.5 ± 1.4	15.36 ± 1.0	0.01 ± 1.44^b^
	90 °C	acetone	63.62 ± 0.5	2.67 ± 0.2	17.70 ± 1.5	16.4 ± 1.6	64.83 ± 0.8	2.21 ± 0.0	17.9 ± 0.3	17.26 ± 0.3	0.84 ± 1.56^a^
		[C_4_mim][BF_4_]	66.95 ± 0.0	−0.47 ± 0.0	14.72 ± 0.1	16.5 ± 0.0	67.59 ± 1.2	−0.60 ± 0.0	15.1 ± 0.2	17.31 ± 0.9	0.76 ± 0.91^a^

a L* = Luminosity; a* = red-green chromaticity. b* = Yellow-blue chromaticity; ΔE* = represents the distance between three colors in the L*, a*, b* space; Δ*E* differential = variation between final-initial. Values with different letters (a–b) in the same evaluated medium (oil and aqueous) are significantly different (*p* < 0.05).

The colorimetric parameters obtained before and after exposure to light indicated significant variations in stability. In the aqueous medium, the largest Δ*E* differentials were observed when acetone was used as the solvent, both in the presence and absence of light, indicating more pronounced color degradation, which suggests that [C_4_mim]­[BF_4_] contributes to the protection of the pigments. In the oily medium, all the treatments showed more pronounced differences in ΔE*, especially under light exposure. However, in the dark, the pulp extracted with [C_4_mim]­[BF_4_] showed a positive differential, suggesting a possible protective effect of the ionic liquid in the oily medium in the absence of light.

The thermal stability of the extract was also evaluated at 60 and 90 °C. In the aqueous medium, the stability of the extract showed slight variations in Δ*E*, with better color preservation at 60 °C for the system with [C_4_mim]­[BF_4_]. At 90 °C, the ionic liquid continued to promote greater stability, while acetone resulted in more degradation of the extract. In the oily medium, the variation in Δ*E* was greater with acetone at 60 °C, revealing the extract’s sensitivity to heat. At 90 °C, both solvents showed similar and positive variations, indicating that, in this medium and at high temperature, color degradation occurs more equally between the solvents.

Overall, the results show that the ionic liquid [C_4_mim]­[BF_4_] was more efficient than acetone in preserving the color of the tucumã extract, especially in aqueous medium and under conditions of lower stress (absence of light or moderate temperatures). These data corroborate previous studies that highlight the potential of ionic liquids as green solvents and stabilizers in the extraction of natural pigments.^28^

### 3.5 Toxicity Assay Using *A. salina*

Tests conducted with *A. salina* are widely used as a reliable tool for assessing the general toxicity of biological samples.^55^ In these tests, parameters are used to classify sample toxicity: LC_50_ values above 1000 μg/mL indicate low toxicity; values between 100 and 500 μg/mL indicate moderate toxicity and values below 100 μg/mL are indicative of high toxicity.^56^

In the results, all tested samples exhibited low toxicity against *A. salina* larvae, as shown in Table 4. Among the evaluated samples, the extract obtained with acetone showed the highest activity, with an LC_50_ value of 682.32 μg/mL after 24 h of exposure. This result showed a statistically significant difference compared to the other extracts, considering the respective confidence intervals. The ionic liquid [C_4_mim]­[BF_4_] exhibited low toxicity in the *A. salina* assay, showing no significant lethal effects. These results suggest that this solvent, can be safely used without notable risk. Lapachol, used as the positive control, displayed high toxicity, with an LC_50_ of 33.51 μg/mL.

**Table 4:** Activities Toxics of Extracts of Fruit Tucumã and β-Carotene against *Artemia salina* 24 Horas of Exposure^a^

samples	LC_50_ ± SD	95% fiducial limits (LCL−UCL)	slope ± SE	*X* ^2^
extract acetone	682.32 ± 0.3^a^	516.21–728.32	1.72 ± 0.1	1.05
extract [C_4_mim][BF_4_]	>1000^b^	824.23–1095.28	1.35 ± 0.1	1.38
β-carotene	>1000^b^	800.54–1063.25	1.32 ± 0.1	1.37
control	33.51 ± 0.3^c^	26.98–48.97	1.10 ± 0.1	1.12

a Means followed by different letters are significantly different (*p* < 0.05). Control = Lapachol LC_50_ lethal concentration for 50% of larvae, LC_90_ lethal concentration for 90% of larvae, SD standard deviation (triplicates), 95% CI confidence interval of 95%, LCL lower confidence limit, UCL upper confidence limit Chi-squared value (*X* ^2^), * extract with the strongest toxic effect.

As reported, larvae exposed to the yellow dye showed lower mortality than those exposed to the red dye, supporting our findings and indicating lower toxicity associated with the yellow dye under the evaluated conditions.^57^ Assessments using *A. salina* have proven to be sensitive and effective indicators of toxicity in bioassays, and are widely employed as a preliminary parameter in the screening of biological activities, with a consistent correlation between observed toxicity and various pharmacological activities, including antitumor, antibacterial, antifungal, and insecticidal effects.^56,58^

### 3.6 Prediction of Biological Activities Using PASS Software

Based on the predictive analysis using PASS (Table 5), β-carotene and α-carotene showed high probabilities of antioxidant activity (Pa = 0.91 and 0.83, respectively), anti-inflammatory activity (Pa = 0.71 and 0.70), and free radical scavenging activity (Pa = 0.83 and 0.78). Lutein demonstrated potential for anti-inflammatory, antihepatotoxic, and cardioprotective activities, with Pa values above 0.6 and Pi values (probability of inactivity) below 0.5. In all evaluated cases, Pa values were higher than Pi values, reinforcing the feasibility of the predicted pharmacological activities. Literature reports further confirm the biological activities of β-carotene, α-carotene, and lutein, supporting the reliability of the PASS predictions. The functions of carotenoids can be understood as essential roles that these compounds play under certain conditions, so that their absence may lead to physiological impairment. Studies indicate that carotenoids exert effects related to the control of gene expression, the regulation of cellular communication and growth, as well as the modulation of enzymes involved in xenobiotic metabolism. It is important to highlight, however, that these biological actions do not occur in isolation, but rather in an integrated and interconnected manner, jointly contributing to the maintenance of cellular balance and proper functioning.^59^

**Table 5:** Pass Prediction Activity of α-Carotene, β-Carotene and Lutein^a^

	activity									
samples	antioxidant		free radical scavenging		antiinflammatory		cardioprotectant		hepatoprotectant	
	Pa	Pi	Pa	Pi	Pa	Pi	Pa	Pi	Pa	Pi
β-carotene	0.91	0.42	0.83	0.46	0.71	0.36	0.57	0.24	0.53	0.13
α-carotene	0.81	0.39	0.78	0.40	0.70	0.33	0.62	0.27	0.45	0.11
lutein	0.52	0.21	0.56	0.12	0.82	0.20	0.81	0.37	0.87	0.21

a Probability to be active (Pa). Probability to be inactive (Pi).

### 3.7 Preliminary Techno-Economic Assessment

Although ionic liquids traditionally present a higher initial cost compared with conventional organic solvents, preliminary techno-economic considerations indicate that their application may become economically advantageous when solvent recyclability and reuse are taken into account. In general, ionic liquids exhibit high chemical stability and can be reused over multiple extraction cycles without significant loss of performance.^60^ Studies conducted by de Souza Mesquita et al.,^27^ Zhou et al.^61^ and Hou et al.^62^ corroborate this evidence, demonstrating that ionic liquids can be employed for five to ten consecutive extraction cycles while maintaining their extraction efficiency. This characteristic reduces the effective cost of the solvent per unit of product, strengthening its economic viability in the extraction of bioactive compounds.

In the present study, [C_4_mim]­[BF_4_] achieved an extraction yield 38% higher than that obtained with acetone, increasing the amount of β-carotene recovered per batch and contributing to greater process productivity. In addition, the greater stability of the extract obtained with the ionic liquid reduces degradation losses, further improving the technical and economic performance of the process.

Although a complete techno-economic assessment is beyond the scope of the present study, factors such as energy demand, solvent recovery efficiency, capital expenditure (CAPEX), operational costs (OPEX) and the minimum selling price of the extracted β-carotene should be examined in future work. Such analyses will be essential to bridge the gap between laboratory-scale optimization and the conditions required for industrial implementation.

## 4 Conclusions

This study demonstrated that ionic liquids can be used as an efficient and sustainable alternative to traditional organic solvents for the extraction of β-carotene from tucumã pulp (*A. aculeatum*), a species native to the Amazon. The [C_4_mim]­[BF_4_], combined with ultrasound-assisted extraction and the central composite rotational design (CCRD), significantly increased the extraction yield while preserving the functional properties of the carotenoid. Furthermore, it exhibited greater thermal and light stability, along with low toxicity, features that are essential for industrial applications. Predictive analysis using PASS (*Prediction of Activity Spectra for Substances*) indicated a high probability of antioxidant, anti-inflammatory, and free radical scavenging activities for the extracted β-carotene, reinforcing its biological potential. These results highlight the importance of Amazonian biodiversity as a source of bioactive compounds and the potential for their valorization through sustainable and efficient extraction methodologies.

## Supplementary Material



## Notes

Analyses of natural dye degradation in different environments: analysis of β-carotene degradation extracted with acetone and [C_4_mim]­[BF_4_] under light exposure, in aqueous and oily media (Figure S1); assessment of β-carotene degradation extracted with acetone and [C_4_mim]­[BF_4_] under dark conditions in aqueous and oily systems (Figure S2); investigation of the thermal stability of β-carotene extracted with acetone and [C_4_mim]­[BF_4_] at 60 °C in both aqueous and oily environments (Figure S3); analysis of the thermal degradation of β-carotene extracted with acetone and [C_4_mim]­[BF_4_] at 90 °C, in aqueous and oily media (Figure S4) (PDF)

## Abbreviations Used

[C_4_mim]­[BF_4_]: 1-butyl-3-methylimidazolium tetrafluoroborate
*K*_d_: degradation constant
*t*_1/2_: longer half-life
*L**: lightness
*a**: red-green chromaticity
*b**: yellow-blue chromaticity
Δ*E**: represents the distance between two colors in the *L**, *a**, *b** color space
CCRD: central composite rotational design
