# Supplementary material for: Optimization of β-Carotene Extraction from Tucumã Fruit (Astrocaryum aculeatum) Using Ionic Liquids: Evaluation of Efficiency, Thermal and Light Stability
Source: ACS Omega. 2025 Dec 18;10(51):63644–54. doi: 10.1021/acsomega.5c11477 (PMC12756795; doi:10.1021/acsomega.5c11477)
Supplement: Supplementary file 1 [file ao5c11477_si_001.pdf]

SUPPORTING INFORMATION

Optimization of β-Carotene Extraction from Tucumã Fruit (*Astrocaryum aculeatum*) Using Ionic Liquids: Evaluation of Efficiency, Thermal and Light Stability

Anne Caroline Gouvêa Ferreira[a,b], Bruna Ribeiro de Lima[a], Wallice Luiz Paxiúba Duncan[c], Leandro Pereira França[a], Jaime Paiva Lopes Aguiar[a] and Francisca das Chagas do Amaral Souza[a,b*]

[a]Laboratory of Functional Analysis and Food Chemistry, National Institute for Amazonian Research (INPA), 69067-375, Manaus, Amazonas, Brazil.

[b]Postgraduate Program in Agriculture in the Humid Tropics, National Institute for Amazonian Research (INPA), 69067-375, Manaus, Amazonas, Brazil.

[c]Laboratory of Functional Morphology, Federal University of Amazonas (UFAM), 69077-000, Manaus, Amazonas, Brazil.

*Corresponding author: E-mail address: francisca.souza@inpa.gov.br (F.C.A. Souza), Phone: + 55 92 99259-1025.

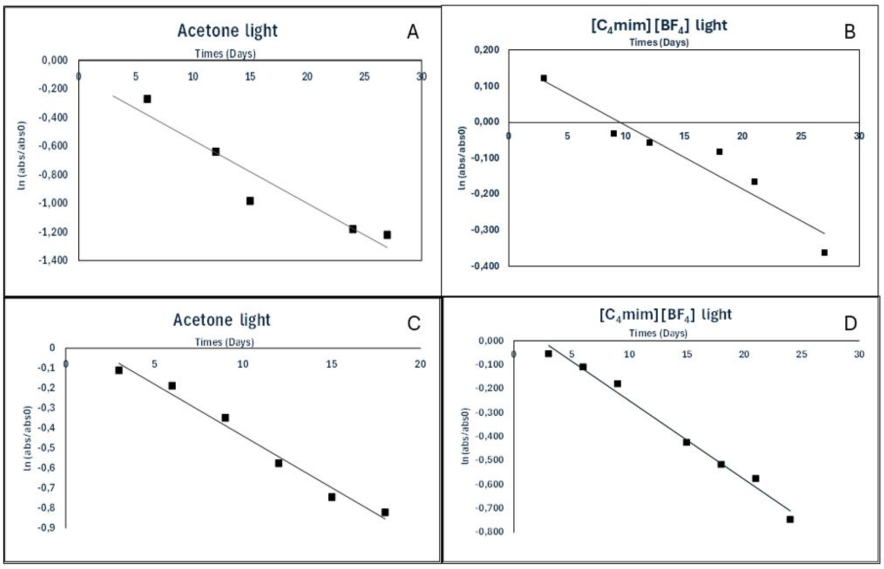

Figure S1 - Analysis of β-carotene degradation extracted with acetone (A and C) and [C_4mim][BF_4] (B and D) under light exposure, in aqueous and oily media, respectively.

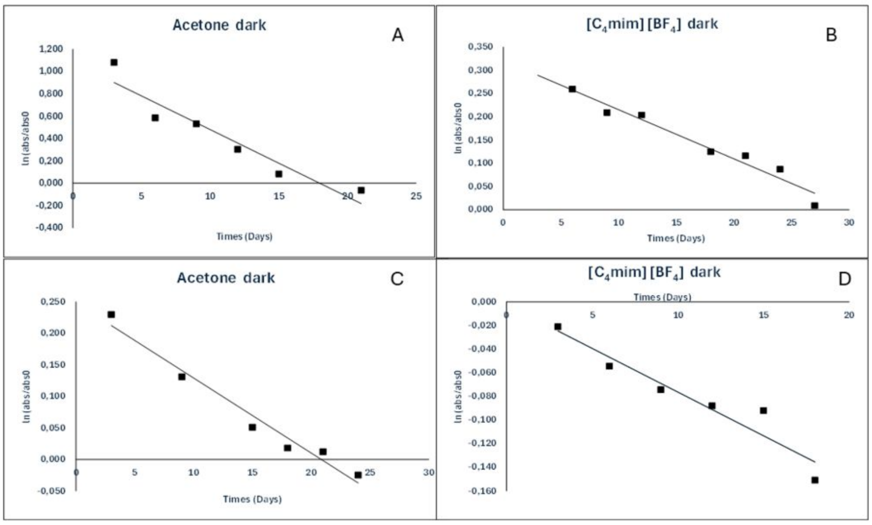

Figure S2- Analysis of β-carotene degradation extracted with acetone (A and C) and [C_4mim][BF_4] (B and D) under dark conditions, in aqueous and oily media, respectively.

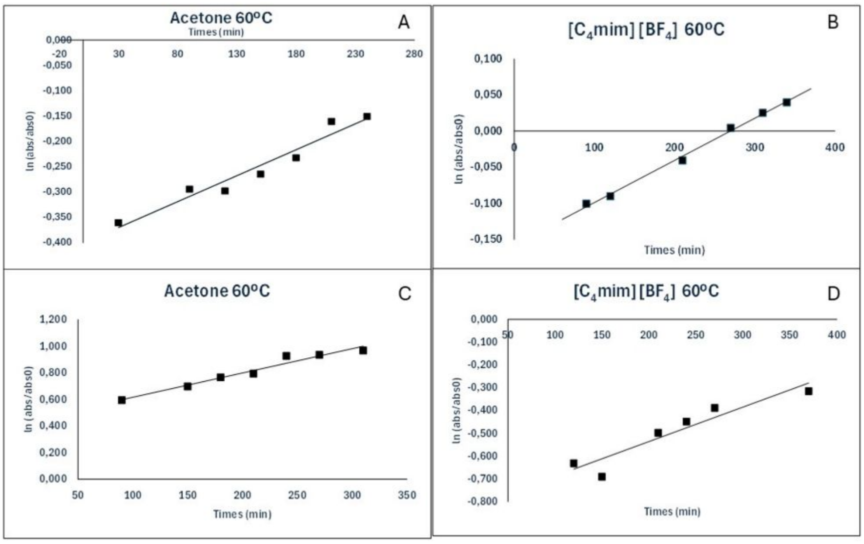

Figure S3- Analysis of the thermal degradation of β-carotene extracted with acetone (A and C) and [C_4mim][BF_4] (B and D) at 60°C, in aqueous and oily media, respectively.

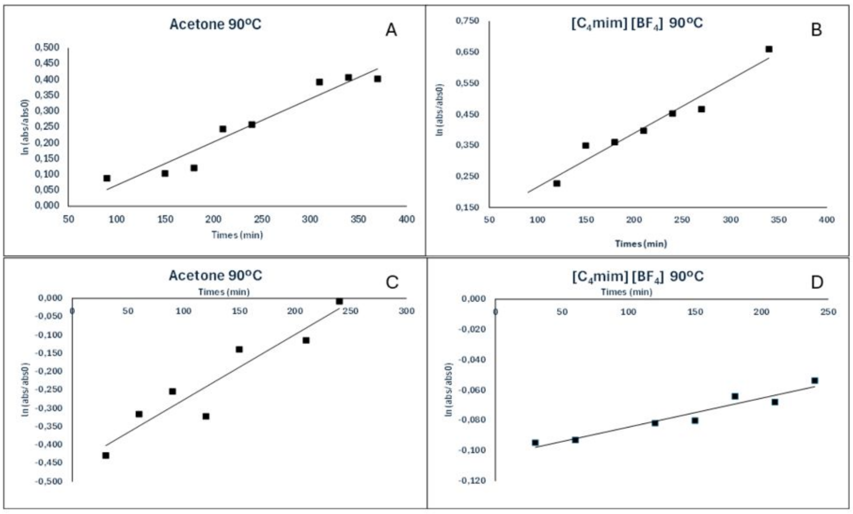

Figure S4 – Analysis of the thermal degradation of β-carotene extracted with acetone (A and C) and [C_4mim][BF_4] (B and D) at 90°C, in aqueous and oily media, respectively.
